# Decoding and vocabulary improvements mediate sustained gains in reading comprehension: Evidence from a randomised controlled trial of a multicomponent reading intervention

**DOI:** 10.1111/jcpp.70159

**Published:** 2026-04-30

**Authors:** Cameron Downing, Catherine Clark, Gwennant Evans, Rachel Cartin, Joseph Smith, Charles Hulme, Manon Jones

**Affiliations:** ^1^ Department of Education University of York York UK; ^2^ Miles Dyslexia Centre Bangor University Bangor UK; ^3^ Department of Psychology Bangor University Bangor UK; ^4^ Department of Education University of Oxford Oxford UK; ^5^ School of Psychology Oxford Brookes University Oxford UK

**Keywords:** Reading comprehension, decoding, intervention, longitudinal mediation, struggling readers, literacy development

## Abstract

**Background:**

Reading comprehension is critical for academic success, yet many children with persistent decoding difficulties struggle to achieve it. This study examined whether a multicomponent literacy intervention is effective in improving reading comprehension and whether any gains in comprehension are mediated by improvements in word reading and vocabulary knowledge.

**Methods:**

In a randomised controlled trial (RCT), 285 English‐speaking children aged 7–9 years with reading difficulties were assigned to a waitlist control group or the Research Informed Literacy with Language (RILL) intervention, a structured, multicomponent programme targeting decoding and language skills. Literacy outcomes were assessed at baseline (t1), postintervention (t2) and at 4‐month follow‐up (t3). The trial was preregistered; https://www.isrctn.com/ISRCTN18940975.

**Results:**

Children receiving RILL showed significantly greater gains in word‐level literacy (*d* = 0.19, *p* < .001), taught vocabulary (*d* = 0.30, *p* = .017) and reading comprehension (*d* = 0.23, *p* = .011) immediately postintervention. Effects were sustained at follow‐up (word‐level literacy *d* = 0.17; taught vocabulary *d* = 0.30; comprehension *d* = 0.25). Mediation analyses, showed a significant indirect effect of the intervention on comprehension at delayed follow‐up via word‐level literacy at t2 (y‐standardised indirect *β* = .10, 95% CI [0.06, 0.12]), with a negligible direct effect (y‐standardised *β* = .01, 95% CI [−0.20, 0.20]). In an additional exploratory parallel‐mediation model, both t2 word‐level literacy and taught vocabulary showed unique indirect effects on t3 comprehension (word‐level literacy indirect: *β* = .16, 95% CI 0.10, 0.21, and taught vocabulary indirect *β* = .11, 95% CI 0.02, 0.23).

**Conclusions:**

Our intervention produced immediate and sustained improvements in word‐level literacy, taught vocabulary and reading comprehension in struggling readers. Persisting decoding weaknesses are common in later primary years, and our findings show that improving word reading can produce enduring benefits for comprehension.

Reading comprehension is a foundational academic skill that underpins learning across subject areas and predicts long‐term educational success (Snow, 2002). The Simple View of Reading (SVR; Gough & Tunmer, 1986) and the Reading is Language model (RiL; Snowling & Hulme, 2025) provide influential frameworks for understanding how comprehension develops. Both models propose that reading comprehension reflects the combined contributions of word decoding and oral language comprehension. A substantial body of evidence supports the predictive roles of both components across languages, including English (Catts, Adlof, & Weismer, 2006; Keenan, Betjemann, & Olson, 2008; Salceda, Alonso, & Castilla‐Earls, 2014) and more transparent orthographies such as Dutch, Finnish, Greek and Norwegian (Caravolas et al., 2019; de Jong & van der Leij, 2002; Florit & Cain, 2011; Kendeu, Papadopoulos, & Kotzapoulou, 2013; Lervåg, Hulme, & Melby‐Lervåg, 2018; Müller & Brady, 2001).

Cross‐linguistic research highlights the role of orthographic depth in shaping this balance. In opaque orthographies such as English, decoding is relatively difficult to master and strongly predicts comprehension in the early years, whereas in transparent orthographies decoding is mastered earlier, allowing language comprehension to play a larger role from a younger age (Caravolas, Lervåg, Defior, Seidlová Málková, & Hulme, 2013; Caravolas et al., 2019; Florit & Cain, 2011; García & Cain, 2014; Leppänen et al., 2004; Verhoeven, van Leeuwe, & Vermeer, 2011; Wimmer & Goswami, 1994). Even in English, however, this balance shifts developmentally: decoding constrains comprehension early on, but as fluency develops, language comprehension becomes the primary driver (Adlof, Catts, & Lee, 2010; Kim, Wagner, & Foster, 2011; Tilstra, McMaster, Van den Broek, Kendeou, & Rapp, 2009).

For struggling readers, this trajectory is disrupted. Persistent decoding difficulties limit fluency and restrict the cognitive resources available for higher‐order comprehension processes (Leach, Scarborough, & Rescorla, 2003; Ouellette & Beers, 2010). Longitudinal research shows that decoding difficulties often persist into later grades, constraining comprehension longer than for typically developing peers (Catts, 2003; Torppa et al., 2007). Decoding also remains a significant predictor of comprehension growth in poor readers after it ceases to be predictive in more skilled readers (Lervåg & Hulme, 2010). Lervåg et al. (2018) further demonstrated a curvilinear relationship, with decoding exerting the strongest effects for children with the lowest skills and weakening once sufficient accuracy and fluency are achieved.

These findings point to decoding as a developmental bottleneck for poorer readers and raise the question of whether intervention‐induced gains in decoding can unlock later gains in comprehension, as predicted by the SVR and RiL models. While phonics‐based interventions reliably improve word reading (Ehri et al., 2001; Hatcher, Hulme, & Ellis, 1994; Torgesen et al., 2001), effects on reading comprehension are mixed and often modest or delayed (Clarke, Snowling, Truelove, & Hulme, 2010; Fricke, Bowyer‐Crane, Haley, Hulme, & Snowling, 2013). This has led to calls for multicomponent programmes targeting both decoding and comprehension (Silverman, Johnson, Keane, & Khanna, 2020). Meta‐analyses indicate that decoding instruction exerts the strongest influence on comprehension outcomes, even after accounting for other skills (Melby‐Lervåg & Lervåg, 2014), and recent trials identify decoding as a key mediator of programme effects (Lovett et al., 2022; Vousden et al., 2022), though effects are attenuated in older children.

Despite this evidence, most findings remain correlational. Longitudinal studies show that early decoding predicts later comprehension (e.g. Adlof et al., 2010; Catts et al., 2006; Lervåg, 2021; Lervåg et al., 2018), but cannot establish causality. Mediation analyses embedded in intervention studies offer a stronger test of mechanisms of change (Hulme, Bowyer‐Crane, Carroll, Duff, & Snowling, 2012), yet few have used temporally lagged designs. Ahmed, Miciak, Taylor, and Francis (2022) examined changes in relations among component skills but measured mediators only at baseline. Fricke et al. (2013) demonstrated a lagged indirect effect via language gains, whereas Vousden et al. (2022) approximated lagged mediation using earlier literacy gains to predict later national outcomes. Roberts, Vaughn, Fletcher, Stuebing, and Barth (2013) modelled growth but did not test mediation. Temporally lagged mediation within an RCT therefore provides a particularly stringent test of whether decoding gains drive later comprehension growth.

Embedding such designs also provides a strong test of theory. The RiL model (Snowling & Hulme, 2025) proposes that later comprehension depends on earlier word reading and language skills, yet to our knowledge, no intervention study has embedded a temporally lagged mediation design to test whether immediate postintervention decoding gains lead to later comprehension improvements.

## The current study

We conducted a randomised controlled trial (RCT) evaluating the Research Informed Literacy with Language (RILL) intervention in struggling readers aged 7–9 years. RILL combines structured decoding instruction with comprehension and language activities. We asked whether RILL produced immediate (t2) and sustained (t3) gains in decoding, vocabulary and comprehension, and whether later comprehension gains were mediated by earlier improvements in decoding.

## Method

### Design and participants

Twenty‐six schools were recruited to participate in the study. In these UK schools 1,152 children in Years 3 and 4 were screened for eligibility using ReadingScreen, an app‐based assessment of word and nonword reading (https://oxedandassessment.com/readingscreen/). The 12 lowest‐performing readers in each school (six in each class) were randomly assigned within classrooms to the intervention (RILL; *n* = 145; mean age = 100.82 months; *SD* = 9.98) or a wait‐list control condition (*n* = 140, mean age = 101.11 months; *SD* = 7.57). Children were selected as the lowest‐performing readers within each class and as a result, at baseline (t1), participants' word reading was substantially below age expectations. On the WRAT‐5 Word Reading subtest (normed mean = 100, *SD* = 15), the sample had a mean standard score of *M* = 64.20 (*SD* = 9.67, range = 55–97). Eighty‐two per cent of the sample scored ≤85 (≥1 *SD* below the normative mean), and 73% scored ≤70 (≥2 *SD*s below the normative mean), indicating that a majority of participants were in the low‐achievement range.

**Figure 1:**
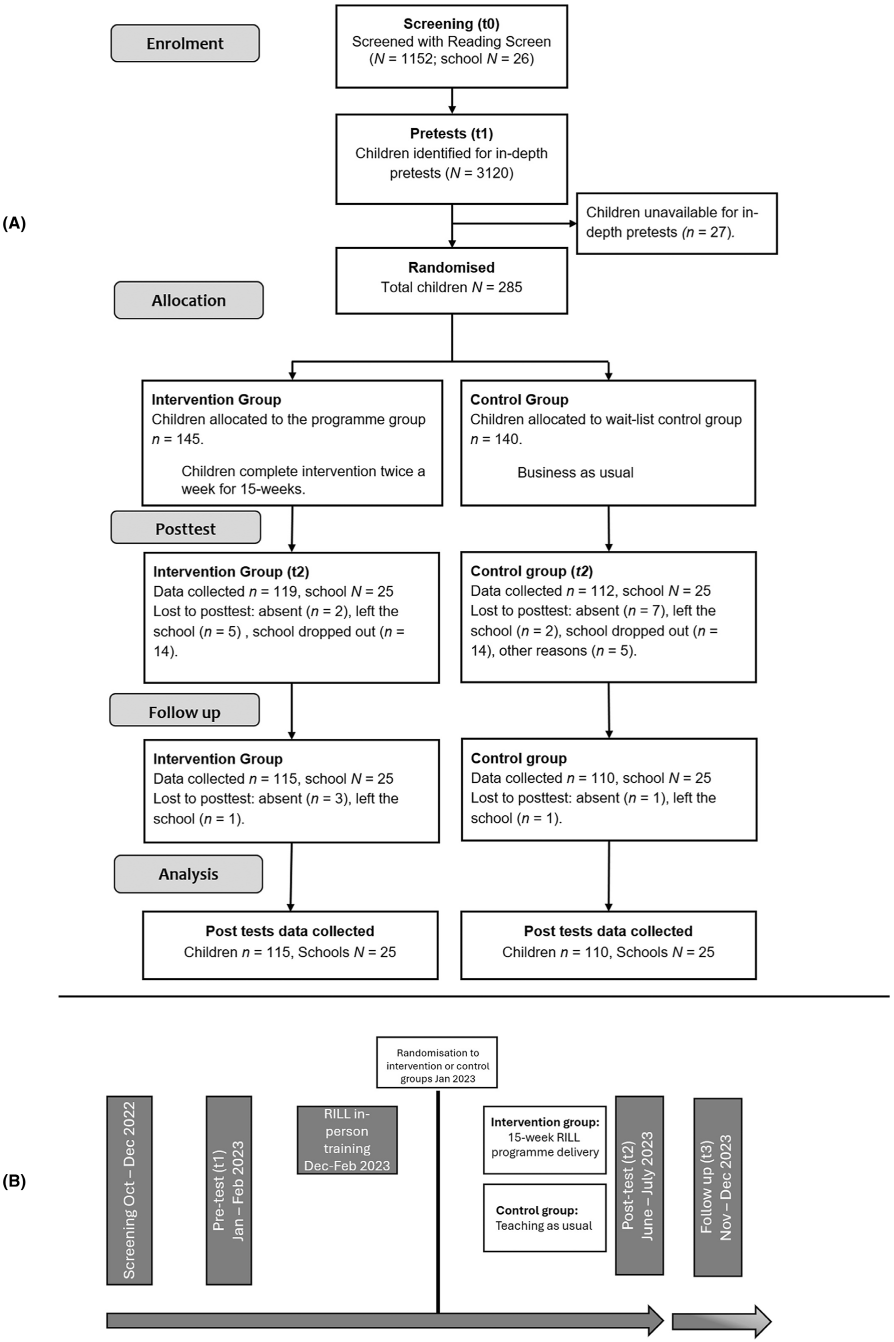
Panel A. CONSORT diagram showing the flow of participants through the RCT. Panel B. Timeline of the trial indicating the times of assessment, training and intervention

Randomisation was carried out by an independent statistician. Headteachers provided consent for school participation, and school administrators distributed invitation letters to caregivers to request consent for their child to participate. Pretest assessments (t1) of reading and related language skills were administered via the platform Gorilla (Anwyl‐Irvine, Massonnié, Flitton, Kirkham, & Evershed, 2020). Posttest assessments (t2) were completed 15 weeks later after the intervention group had completed the programme, and the follow‐up assessment (t3) was conducted 4 months later. Participant flow is shown in the CONSORT diagram (Figure 1a; Schulz, Altman, & Moher, 2010). Figure 1b provides a timeline for the project. See also our preregistration of the trial here: https://www.isrctn.com/ISRCTN18940975. Testing was done on site during school hours. During data collection, we experienced technical issues in collecting planned language measures; this led to us dropping a hypothesis about the effects of the intervention on language. However, we were still able to collect expressive vocabulary measures, and so we include analysis of the effects of the intervention on this measure as an exploratory analysis. No other changes to methods were made following trial registration, and the trial end was as planned.

### The RILL intervention

The RILL intervention (Research Informed Literacy with Language; Downing et al., 2025) is a 15‐week, twice‐weekly, individualised programme (totalling 20 h). It builds on previously validated interventions, including REVI (Duff et al., 2008) and Reading Intervention (Bowyer‐Crane et al., 2008; Hatcher et al., 1994; Hatcher, Hulme, & Snowling, 2004), with a focus on phonological awareness, vocabulary and decoding. Lessons, delivered online by trained teaching assistants (TAs), incorporate distributed learning principles (Latimier, Peyre, & Ramus, 2021).

RILL was designed to strengthen core literacy skills in children aged 7–9 years with persistent reading difficulties, particularly vocabulary and word‐level decoding skills that underpin comprehension (Scarborough, 2001). Teaching Assistants (TAs) delivered the RILL lessons twice a week for 15 weeks in small groups in school (2–3 children at a time). The TAs led the small group through interactive activities presented on a computer.

Each 40‐min lesson follows a structured sequence:

1. Words of the Day (5 min): Children are taught the meanings of 2 Tier 2 vocabulary words using definitions, visual support and discussion (Beck et al., 1982, 2002).
2. Passage of the Day (10 min): Children read aloud a short passage containing the target words.
3. Word Games (5 min): Activities to support phonemic awareness and phonics (e.g. blending).
4. Word Writing (10 min): Spelling instruction with a focus on vowel patterns and sound‐letter correspondence.
5. Story Time (5 min): Narrative skills practice using scaffolded story creation tasks (Clarke et al., 2010).
6. Recap (5 min): Children reviewed and applied the target vocabulary.

Schools continued to provide business‐as‐usual instruction and support to pupils allocated to the wait‐list control arm and any additional literacy support that pupils were already receiving continued.

### Ethical information

Written informed consent was obtained from parents/caregivers for all participating children, with child assent also sought in accordance with ethical guidelines. The study received ethical approval from the School of Psychology ethics board at Bangor University, granted on 07.09.2022, reference number 2022‐17213.

### Assessment measures

#### Selection measure

The screening tool used to select children for the trial was *ReadingScreen* (https://oxedandassessment.com/readingscreen/). This mobile app containing two subtests: word and nonword reading. The child is asked to read aloud a series of 60 words and 34 nonwords of increasing difficulty. The assessment took less than 10 min to complete per child and was administered, following training, by the class teacher or TA.

#### Primary outcomes

The primary outcome measures are two latent literacy variables reflecting: (1) Word‐level literacy (created from measures of single word reading, single word spelling, word fluency and pseudoword fluency) and (2) Reading Comprehension.

#### Word‐level literacy measures

Word Reading Accuracy (*Wide Range Achievement Test 5* ^*th*^ *edition* WRAT‐5; Wilkinson & Robertson, 2017): This untimed test measures letter identification and word recognition. The child reads each item aloud. The words are of increasing complexity in terms of regularity and word frequency, with a total of 15 letters and 55 words. The task is discontinued following 10 consecutive errors.

Word Reading Accuracy (ReadingScreen) https://oxedandassessment.com/readingscreen/: This untimed test (described above) measures word recognition accuracy. The child reads each item aloud. The words are of increasing complexity in terms of regularity and word frequency, with 60 items in total. Responses are marked as correct or incorrect by the administrator.

Word Spelling Accuracy (WRAT‐5): This test measures the child's ability to write letters and words to dictation without a time limit. Words increase in complexity in terms of spelling patterns with a total of 13 letters and 42 words. The task is discontinued following 10 consecutive errors.

Passage Reading Accuracy (*York Assessment of Reading for Comprehension Passage Reading; YARC Passage Reading*; Snowling et al., 2009): To measure word reading accuracy during passage reading, children read aloud four passages (Levels) of the YARC: at t1, t2 and t3. One point is awarded for each correct word. At each time point, the accuracy scores from each passage were used to produce a passage reading accuracy score. Administering the same four passages to all participants at each time point was designed to capture the full range of ability (c.f., Nation, Cocksey, Taylor, & Bishop, 2010).

#### Reading comprehension measure

YARC Comprehension Questions: (*YARC Passage Reading*; Snowling et al., 2009): To measure reading comprehension, after reading each of the passages aloud (see Passage reading accuracy), children are asked a series of comprehension questions based on the passage. Published scoring guidelines were followed when scoring responses to each of the comprehension questions.

#### Secondary outcome

A short expressive vocabulary task was used to assess knowledge of the vocabulary explicitly taught over the course of the programme. A subset of 18 words drawn from the 60 words taught during the RILL lessons was presented to the child aurally. The child was then required to briefly define the meaning of each word. Children's definitions were scored on a 3 point scale (0—*incorrect, or no response*; 1—*partially correct definition*; 2—*fully correct definition*).

### Procedure

Following written caregiver consent, children completed baseline testing (t1; ~1 h). Teaching Assistants, trained by the research team, administered identical assessments at all time points in school during school hours. Assessments were administered using the Gorilla platform (https://gorilla.sc), with support from research assistants. Posttesting occurred after 15 weeks (t2) and again 4 months later (t3). Assessors were blind to group assignment at t2 and t3, and 15% of all data was rescored to ensure interrater reliability. Intervention began in early 2023.

Implementation fidelity was assessed via a single, structured 45‐min observation per school by a trained Research Assistant. Each lesson component (words of the day, passage of the day, word games, spelling, story writing, revisiting words of the day), as well as overall lesson timing, pupil engagement and use of technology, was rated on a 3‐point scale (1 = *minimal*, 2 = *partial*, 3 = *full implementation*). Observers also recorded qualitative notes, and Teaching Assistants were invited to provide feedback. All schools achieved total scores >20 (out of 27), indicating high fidelity.

### Trial registration

This trial was preregistered with the ISRCTN registry (ISRCTN18940975; registered 20/06/2023; https://www.isrctn.com/ISRCTN18940975).

## Results

Analyses were conducted in Stata® 18.5 (Stata Corporation, College Station, TX, USA, 2023) and structural equation models (SEM) were implemented in Mplus 8.11 using full information maximum likelihood (FIML) estimators to handle missing data. All analyses were performed on an intention‐to‐treat basis. The study was preregistered (https://www.isrctn.com/ISRCTN18940975) and the data and analysis files are available at: https://osf.io/5vmqj/?view_only=92f4538009ca4657ab08975d03b364de.

A total of 1,152 children in Years 3 and 4 were screened with ReadingScreen at t0 across 26 schools. Children were randomly allocated within the school to the intervention or control group (6 children per arm). At posttest (t2), 26 children from the intervention group and 28 children from the control group were lost. At follow‐up (t3), a further 4 children from the intervention group and 2 children from the control group were lost. There were no significant differences at pretest between the children who completed the study and those who were lost in age (*p* = .391, *d* = .13) or ReadingScreen scores (*p* = .937, *d* = .01). Table 1 shows the descriptive statistics for all measures at screening (t0), pretest (t1), posttest (t2) and follow‐up (t3) for both groups. Both groups performed similarly at pretest as expected given random assignment.

**Table 1:** Mean, standard deviations and reliabilities of raw scores for the intervention and control groups for the outcome measures at screening (t0), pretest (t1), posttest (t2) and follow‐up (t3)

	Intervention			Control Group			Reliability
	*N*	*M*	*SD*	*N*	*M*	*SD*	
Age							
t0	145	100.82	9.98	140	101.11	7.57	
Word literacy							
Reading screen							.95^a^
t0	116	16.67	10.26	111	17.72	9.98	
t2	68	24.46	10.74	68	24.73	11.48	
t3	76	26.37	11.64	76	27.12	10.49	
WRAT reading							.95–.96^a^
t1	124	9.48	5.67	122	10.66	5.69	
t2	115	11.59	5.95	107	11.78	6.23	
t3	113	13.64	5.75	106	13.84	6.28	
WRAT spelling							.91–.92^a^
t1	143	5.13	2.80	137	5.18	2.58	
t2	115	6.29	3.52	112	6.03	3.12	
t3	113	7.12	3.34	109	6.82	3.29	
YARC reading accuracy							.85–.93^b^
t1	134	111.44	67.07	124	125.77	67.79	
t2	111	208.29	96.56	102	201.09	97.12	
t3	110	225.09	87.80	105	222.87	86.20	
Reading comprehension							
Passage 1							.55–.69^b^
t1	118	3.03	1.92	115	3.63	1.76	
t2	89	3.93	2.05	89	4.01	2.18	
t3	95	4.13	2.00	95	4.18	2.29	
Passage 2							
t1	118	3.50	2.06	117	4.13	2.31	
t2	95	2.84	2.14	85	2.51	1.92	
t3	96	3.18	1.98	93	3.19	2.24	
Passage 3							
t1	118	2.90	1.73	102	3.38	1.67	
t2	107	3.45	1.71	93	3.66	1.65	
t3	106	3.75	1.71	97	3.77	1.62	
Passage 4							
t1	113	1.46	1.66	100	1.89	1.68	
t2	101	2.24	1.90	86	2.45	1.75	
t3	102	2.84	2.11	97	2.94	1.75	
Language							
Vocabulary 1							.77–.85^b^
t1	119	2.30	1.53	115	2.10	1.72	
t2	105	3.37	2.04	102	2.31	1.97	
t3	110	3.58	2.14	109	2.80	2.20	
Vocabulary 2							
t1	119	1.22	1.55	115	1.26	1.55	
t2	105	2.62	2.28	102	2.02	2.31	
t3	110	2.82	2.35	109	1.93	2.14	
Vocabulary 3							
t1	115	3.47	2.02	115	3.22	2.33	
t2	102	4.66	2.31	102	3.74	2.60	
t3	109	4.88	2.40	109	3.95	2.65	

Reliabilities are reported as Cronbach's alpha.

^a^ Published reliabilities.

^b^ Sample reliabilities.

### Primary outcome effects

Our primary outcomes were word‐level literacy and reading comprehension. Intervention effects were estimated using ANCOVA models in which the relevant latent outcome at posttest (t2; short‐term effects) or follow‐up (t3; longer‐term effects) was regressed on its pretest value (t1) and a dummy‐coded group variable (intervention = 1, control = 0). Prior to these analyses, confirmatory factor analyses (CFAs) were conducted to evaluate model fit and test factorial invariance across time. Residuals for the same indicators were correlated across time points, which improved the model fit. Clustering within schools was accounted for using robust (Huber‐White) standard errors with school as the clustering variable.

For each model, we tested the homogeneity‐of‐slopes assumption by comparing models with group‐specific versus constrained pretest‐outcome slopes. In all cases, the constrained model was supported, indicating no evidence of a group‐by‐pretest interaction; intervention effects did not vary as a function of baseline ability. Intervention effects are reported as y‐standardised regression coefficients for the group variable, equivalent to Cohen's *d*.

#### Immediate effects on word literacy

**Figure 2:**
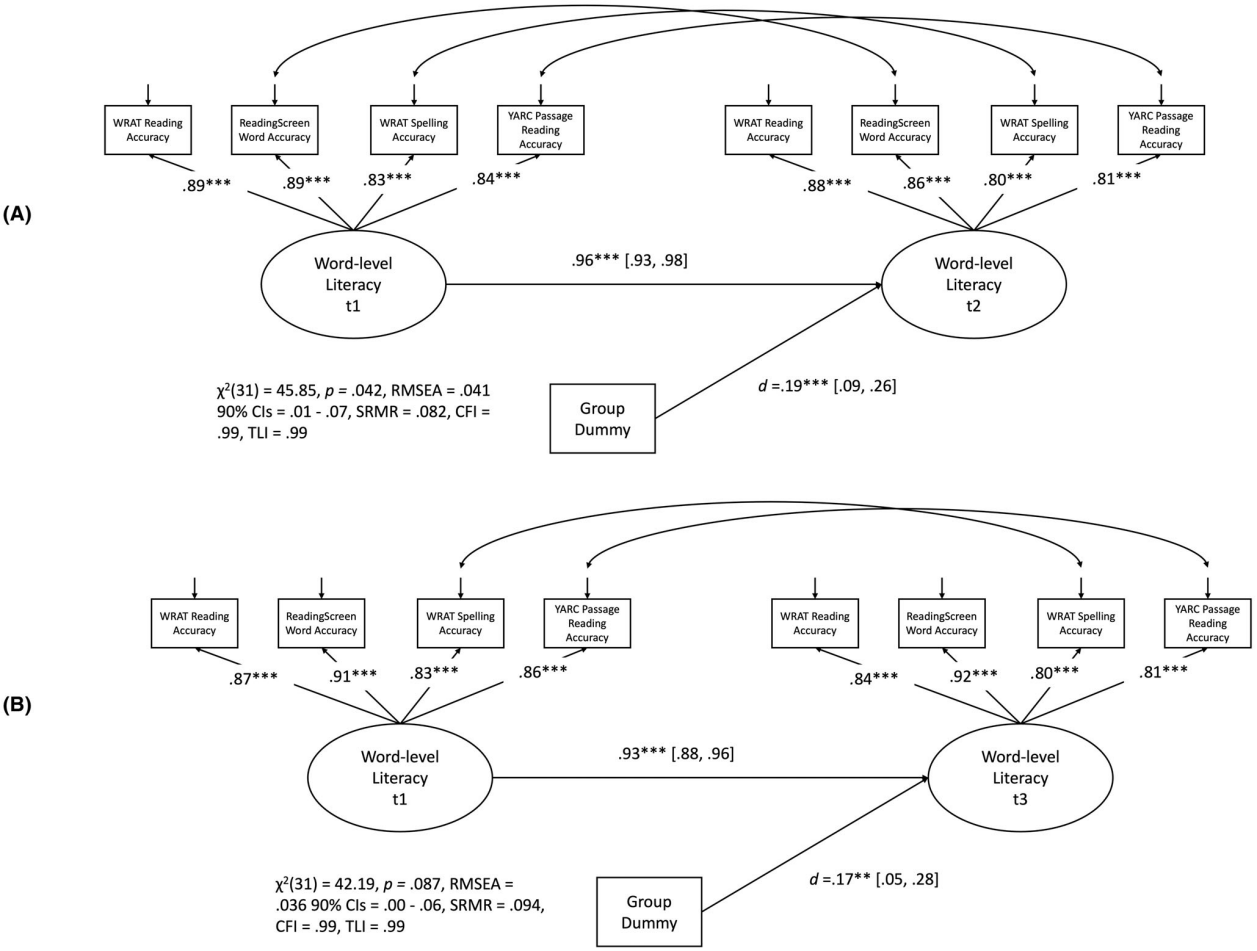
Panel A. Path diagram for latent variables of word‐level literacy at pretest (t1) and posttest (t2). Factor loadings and the autoregressive path are standardised estimates. The effect of the intervention is shown by the path from the group dummy (intervention vs. control) to word‐level literacy at posttest, which is y‐standardised (equivalent to Cohen's *d*). CIs are 95% robust 95% CIs accounting for clustering within schools are reported in square brackets. ***p < .01. Panel B. Path diagram for latent variables of word‐level literacy at pretest (t1) and follow‐up (t3). Factor loadings and the autoregressive path are standardised estimates. The effect of the intervention is shown by the path from the group dummy (intervention vs. control) to word‐level literacy at follow‐up, which is y‐standardised (equivalent to Cohen's *d*). CIs are 95% robust 95% CIs accounting for clustering within schools are reported in square brackets. **p < .01, ***p < .01

The word‐level literacy latent variable was defined using four measures (WRAT Word Reading, WRAT Word Spelling, ReadingScreen Word Reading Fluency and YARC Reading Accuracy). Residuals were correlated across time for all indicators except WRAT Word Reading. Tests of longitudinal invariance supported full configural, metric and scalar invariance (see Appendix S1, Tables S1 and S2), and the scalar model was therefore used. The ANCOVA model (Figure 2, Panel A) showed good fit, *χ* ^2^(31) = 45.85, *p* = .042, RMSEA = 0.041 (90% CI [0.01, 0.07]), SRMR = 0.082, CFI = 0.99, TLI = 0.99. Children in the intervention group made significantly greater gains in word‐level literacy immediately postintervention than controls (*d* = 0.19, 95% CI [0.09, 0.26], *p* < .001).

#### Longer‐term effects on word literacy

Longer‐term effects were examined using word‐level literacy latent variables derived from the four word reading and spelling measures at pretest (t1) and follow‐up (t3). Residuals were correlated for WRAT Word Spelling and YARC Reading Accuracy across time. Tests supported full configural, metric and scalar invariance (see Appendix S1, Tables S1 and S2), and the scalar model was therefore used (Figure 2, Panel B). Model fit was good, *χ* ^2^(31) = 42.19, *p* = .087, RMSEA = 0.036 (90% CI [0.00, 0.06]), SRMR = 0.094, CFI = 0.99, TLI = 0.99. Children in the intervention group showed significantly greater gains in word‐level literacy than controls at follow‐up (*d* = 0.17, 95% CI [0.05, 0.28], *p* = .004), an effect comparable in size to the immediate postintervention effect, indicating maintenance of gains.

#### Immediate effects on reading comprehension

**Figure 3:**
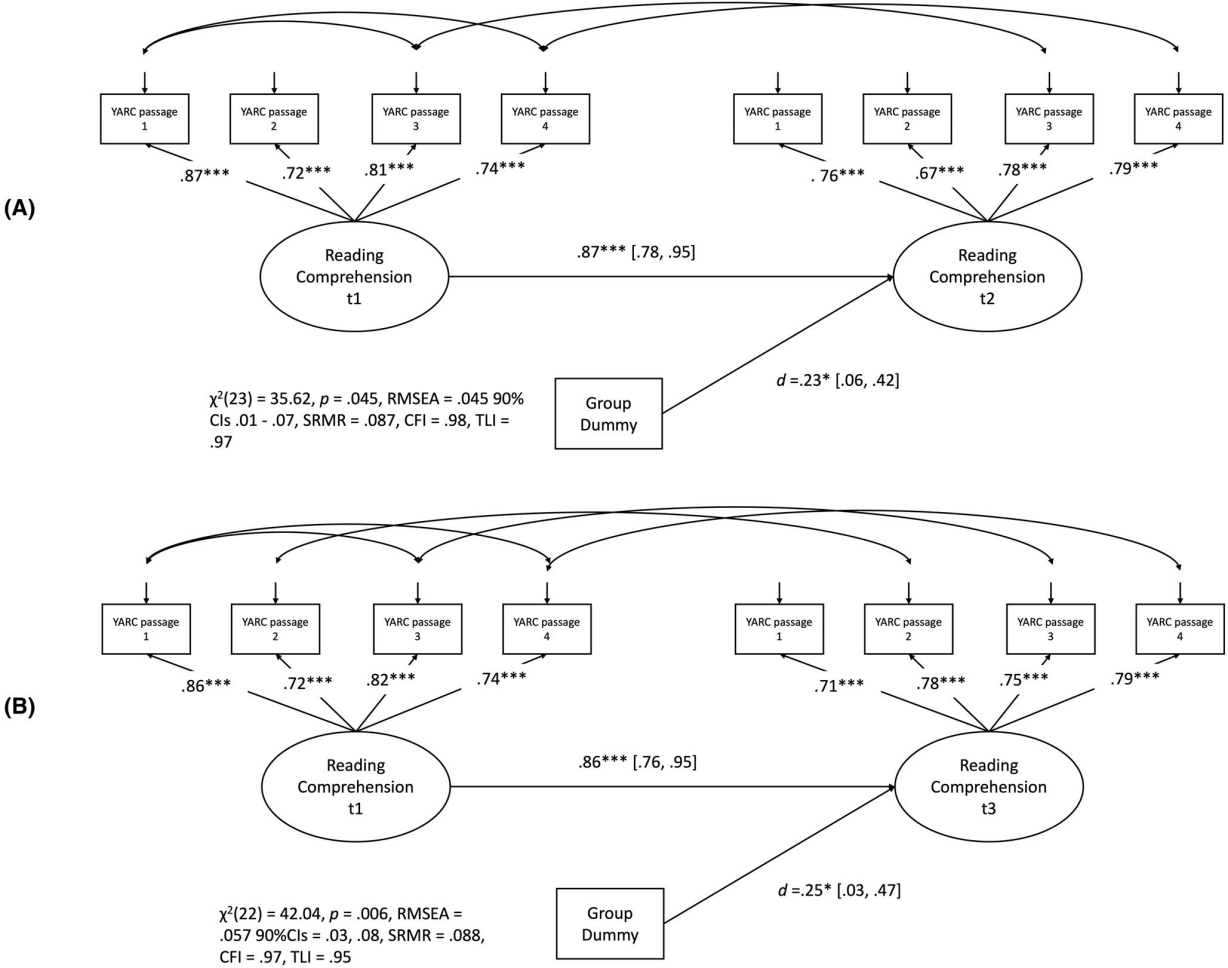
Panel A. Path diagram for latent variables of reading comprehension at pretest (t1) and posttest (t2). Factor loadings and the autoregressive path are standardised estimates. The effect of the intervention is shown by the path from the group dummy (intervention vs. control) to word‐level literacy at posttest, which is y‐standardised (equivalent to Cohen's *d*). CIs are 95% robust 95% CIs accounting for clustering within schools are reported in square brackets. **p < .01, ***p < .01. Panel B. Path diagram for latent variables of reading comprehension at pretest (t1) and follow‐up (t3). Factor loadings and the autoregressive path are standardised estimates. The effect of the intervention is shown by the path from the group dummy (intervention vs. control) to reading comprehension at follow‐up, which is y‐standardised (equivalent to Cohen's *d*). CIs are 95% robust 95% CIs accounting for clustering within schools are reported in square brackets. **p < .01, ***p < .01

The reading comprehension latent variable was derived from comprehension question scores for four YARC passages. Residuals for Passages 3 and 4 were correlated across time, and passages were correlated within the pretest to account for shared task variance and improve model fit. Full configural and partial metric invariance (three noninvariant loadings) were supported (see Appendix S2, Tables S3 and S4). The partial metric model was used to estimate intervention effects. The ANCOVA model (Figure 3, Panel A) was a good fit to the data, *χ* ^2^(23) = 35.62, *p* = .045, RMSEA = 0.045 (90% CI [0.01, 0.07]), SRMR = 0.087, CFI = 0.98, TLI = 0.98. Children in the intervention group showed significantly greater gains in reading comprehension at posttest than controls (*d* = 0.23, 95% CI [0.06, 0.42], *p* = .011).

#### Longer‐term effects on reading comprehension

Longer‐term intervention effects on reading comprehension were tested using latent variables derived from four YARC passages administered at pretest (T1) and follow‐up (T3). Residuals for Passages 2–4 were correlated across time, and passages were correlated within pretest to account for shared task variance. Full configural and partial metric invariance (three noninvariant loadings) were supported (see Appendix S2, Tables S3 and S4), and the partial metric model was therefore used. The ANCOVA model (Figure 3, Panel B) showed an adequate fit, *χ* ^2^(22) = 42.04, *p* = .006, RMSEA = 0.057 (90% CI [0.03, 0.08]), SRMR = 0.088, CFI = 0.97, TLI = 0.95. Children in the intervention group showed significantly greater reading comprehension gains at follow‐up than controls (*d* = 0.25, 95% CI [0.03, 0.47], *p* = .026).

### Secondary outcome effects

As with the primary outcome measures, we measured the effects of the intervention using the ANCOVA approach described earlier. This analysis was not preregistered.

#### Immediate effects on vocabulary

**Figure 4:**
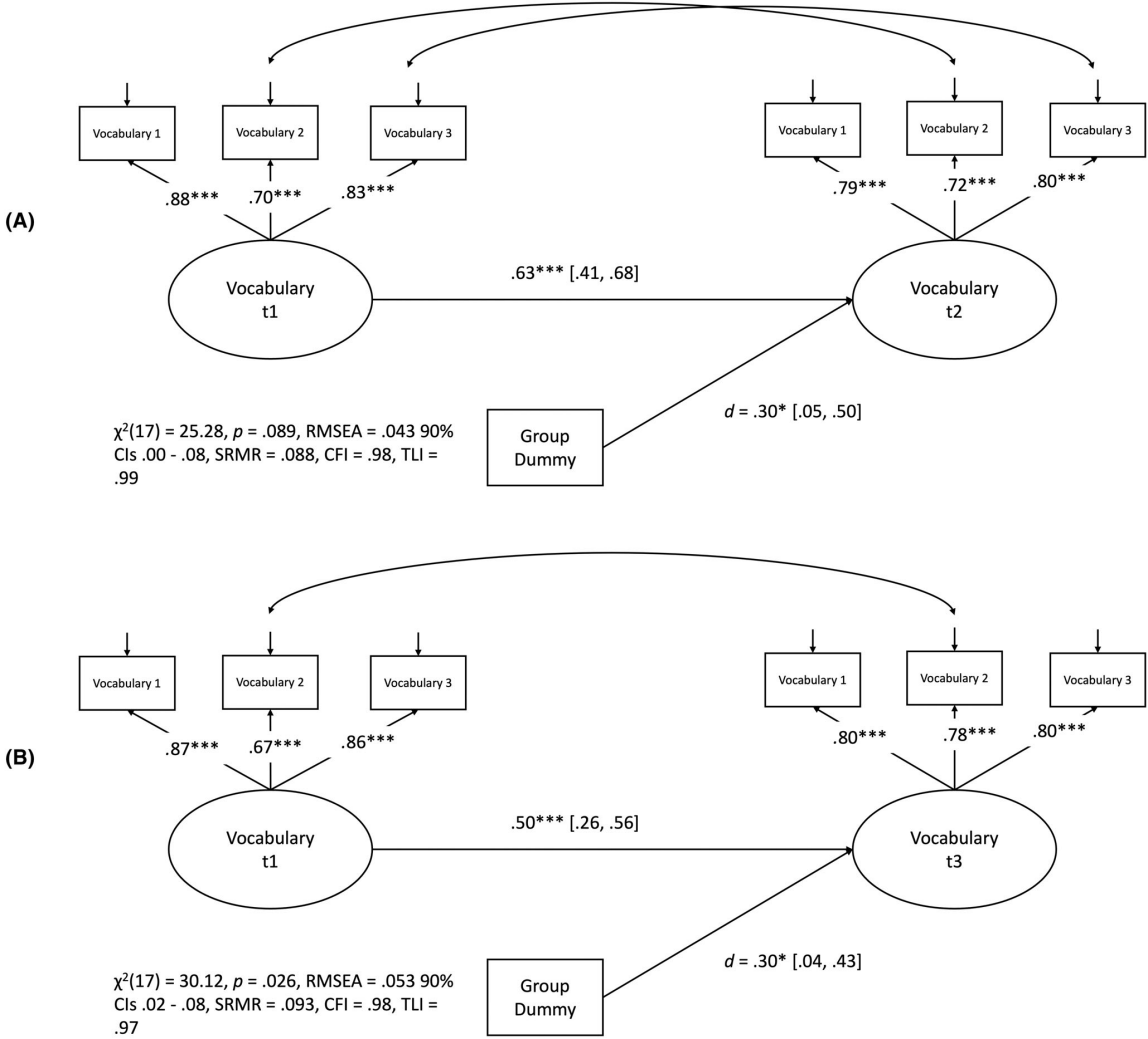
Panel A. Path diagram for latent variables of taught expressive vocabulary at pretest (t1) and posttest (t2). Factor loadings and the autoregressive path are standardised estimates. The effect of the intervention is shown by the path from the group dummy (intervention vs. control) to word‐level literacy at posttest, which is y‐standardised (equivalent to Cohen's *d*). CIs are 95% robust CIs accounting for clustering within schools are reported in square brackets. **p < .01, ***p < .01. Panel B. Path diagram for latent variables of expressive vocabulary at pretest (t1) and follow‐up (t3). Factor loadings and the autoregressive path are standardised estimates. The effect of the intervention is shown by the path from the group dummy (intervention vs. control) to reading comprehension at follow‐up, which is y‐standardised (equivalent to Cohen's *d*). CIs are 95% robust CIs accounting for clustering within schools are reported in square brackets. **p < .01, ***p < .01

The expressive vocabulary latent variable was defined using three parcels (six words per parcel) at each time point. Residuals were correlated across time for all parcels except Parcel 1. Full configural, metric and scalar invariance was supported (see Appendix S3, Tables S5 and S6), and the scalar model was used to estimate intervention effects. The ANCOVA model (Figure 4, Panel A) showed good fit, *χ* ^2^(17) = 25.28, *p* = .089, RMSEA = 0.043 (90% CI [0.00, 0.08]), SRMR = 0.088, CFI = 0.98, TLI = 0.98. Children in the intervention group made significantly greater gains in taught vocabulary than controls at posttest (*d* = 0.30, 95% CI [0.05, 0.54], *p* = .017).

#### Longer‐term effects on vocabulary

Longer‐term effects on expressive vocabulary were examined using latent variables at pretest (T1) and follow‐up (T3). Residuals for Parcel 2 were correlated across time. Full configural, metric and scalar invariance was supported (see Appendix S3, Tables S5 and S6), and the scalar model was used to test group effects. The model (Figure 4, Panel B) showed good fit, *χ* ^2^(17) = 30.12, *p* = .026, RMSEA = 0.053 (90% CI [0.02, 0.08]), SRMR = 0.093, CFI = 0.98, TLI = 0.97. The intervention group showed significantly greater vocabulary gains than controls at follow‐up (*d* = 0.30, 95% CI [0.04, 0.43], *p* = .021), with effects comparable to those observed at posttest, indicating maintenance.

### Testing mediators of gains in reading comprehension

**Figure 5:**
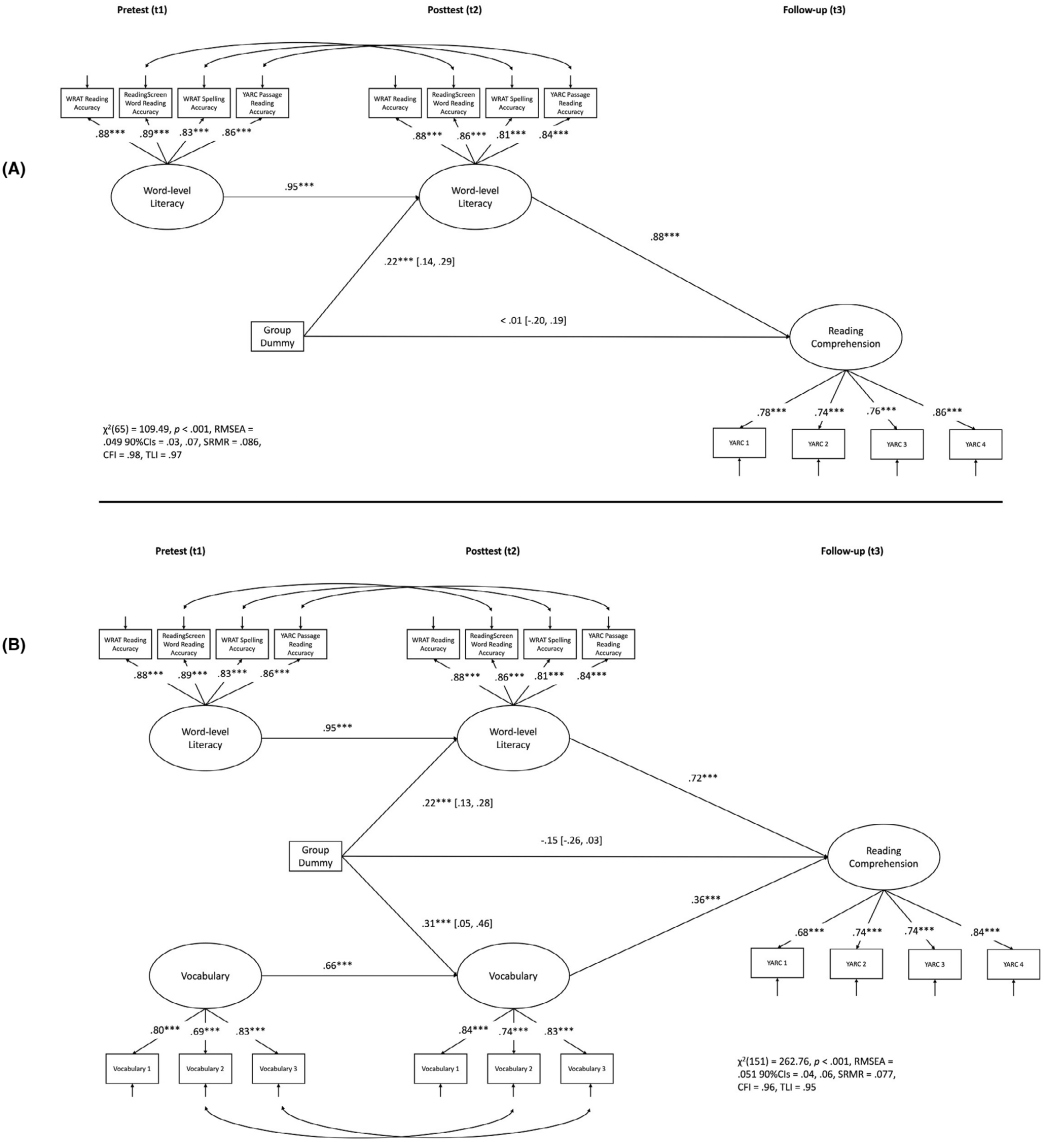
Panel A. Path diagram of the mediation model testing the indirect influence of the intervention on reading comprehension via word‐level literacy. Factor loadings and the autoregressive path are standardised estimates. The effect of the intervention is shown by the paths from the group dummy (intervention vs. control) to word‐level literacy and reading comprehension at follow‐up, which is y‐standardised (equivalent to Cohen's *d*). Panel B. Path diagram of the mediation model testing the indirect influence of the intervention on reading comprehension via word‐level literacy and vocabulary. Factor loadings and the autoregressive path are standardised estimates. The effect of the intervention is shown by the paths from the group dummy (intervention vs. control) to word‐level literacy, vocabulary and reading comprehension at follow‐up are y‐standardised (equivalent to Cohen's *d*). CIs are 95% robust CIs accounting for clustering within schools are reported in square brackets. **p < .01, ***p < .01

We tested whether later reading comprehension gains were mediated by earlier improvements in word‐level literacy using a temporally lagged mediation model. Word‐level literacy at pretest (T1) and posttest (T2) was modelled as a latent construct defined by WRAT Word Reading, WRAT Word Spelling, ReadingScreen word reading and YARC passage reading accuracy, with residuals correlated across time (except WRAT Word Reading). Reading comprehension at follow‐up (T3) was defined by the four YARC passage comprehension scores. The model regressed T1 word‐level literacy and group onto T2 word‐level literacy, and T2 word‐level literacy and group onto T3 reading comprehension (Figure 5a).

The model provided a good fit to the data, *χ* ^2^(65) = 109.49, *p* < .001, RMSEA = 0.049 (90% CI [0.03, 0.07]), SRMR = 0.086, CFI = 0.98, TLI = 0.97. Children in the intervention group showed significantly greater gains in word‐level literacy at posttest than controls (*d* = 0.22). The direct effect of the intervention on reading comprehension at follow‐up was negligible (y‐standardised = 0.01, 95% CI [−0.20, 0.19]); however, the indirect effect via posttest word‐level literacy was significant (standardised indirect effect = 0.10, 95% CI [0.06, 0.12], *p* < .001), equivalent to *d* = 0.19 (Kraft, 2020).

Given the intervention's multicomponent nature, improvements in reading comprehension cou an additional, exploratory parallel‐mediation model including vocabulary at posttest (t2) as a proxy for language skills alongside word‐level literacy to test whether each uniquely mediated follow‐up reading comprehension (t3; Figure 5b). We note that vocabulary captures only one aspect of oral language and that the study was not specifically powered to compare multiple mediators. These analyses are strictly exploratory.

This model provides an appropriate fit to the data, *χ* ^2^(151) = 262.76, *p* < .001, RMSEA = 0.051 90% CI = [0.04, 0.06], SRMR = 0.077, CFI = 0.96, TLI = 0.95. In this model, accounting for later reading comprehension, we found that children who completed the intervention made educationally significantly greater gains in their word‐level literacy (*d* = 0.22) and vocabulary (*d* = 0.31) than children who did not complete the intervention (Kraft, 2020). The direct effect of the intervention on later reading comprehension in this model was nonsignificant (y‐standardised effect = −0.15 95% CI [−0.34, 0.05]). The indirect effect of the intervention on reading comprehension via posttest word‐level literacy was significant (intervention ➔ posttest word‐level literacy ➔ follow‐up reading comprehension; standardised indirect effect = 0.16; 95% CI [0.10, 0.21]; *p* < .001) as was the indirect effect of the intervention on reading comprehension via posttest vocabulary (intervention ➔ posttest vocabulary ➔ follow‐up reading comprehension; standardised indirect effect = 0.11; 95% CI [0.02, 0.23]; *p* = .048).

## Discussion

The present study evaluated a language‐rich reading intervention programme and its effects on both reading accuracy and reading comprehension. A particular focus was whether intervention‐induced gains in decoding could account for later improvements in reading comprehension in children with persistent decoding difficulties.

Consistent with prior multicomponent intervention trials, children who received RILL made significantly greater gains than controls in word decoding, taught vocabulary knowledge and reading comprehension immediately after the programme (t2) with effects maintained at 4‐month follow‐up (t3). Although the sample was selected as the lowest‐performing readers within each class, there was substantial variation in baseline reading ability (including a minority scoring in the average range on standardised word reading). Homogeneity‐of‐slopes tests provided no evidence that intervention effects varied as a function of initial ability.

The mediation analyses help to clarify the mechanisms underlying the sustained improvements in reading comprehension at follow‐up. In the temporally lagged model, the t3 difference in comprehension was statistically accounted for, in part, by earlier gains in word‐level literacy at t2, consistent with the central role of word reading in supporting later comprehension. In an additional exploratory parallel‐mediation model, posttest gains in taught vocabulary knowledge also showed an independent indirect pathway to later comprehension alongside word‐level literacy. However, the indirect pathway via word‐level literacy was stronger than the vocabulary pathway.

It is also important to note that the two mediators differed in their proximity to programme content: the vocabulary factor indexed knowledge of words explicitly taught during RILL (near transfer), whereas the word‐level literacy factor was derived from broader decoding/word‐reading measures. This difference in measurement scope likely contributed to the relatively larger posttest gain in taught vocabulary, while the larger indirect pathway via word‐level literacy reflects the stronger association between general word‐level skill and later reading comprehension in the model.

This pattern reflects the stronger association between word‐level literacy and later comprehension in the model, and suggests that sustained comprehension benefits may be driven primarily by improvements in word‐level skills (Perfetti & Stafura, 2014; Tilstra et al., 2009), with an additional (smaller and more uncertain) contribution from taught vocabulary knowledge. Together, the findings are consistent with multicomponent accounts of reading development in which both word‐level and language‐related learning support comprehension, but with word‐level constraints remaining particularly influential in struggling readers (Gough & Tunmer, 1986; Snowling & Hulme, 2025).

These findings extend the Simple View of Reading (Gough & Tunmer, 1986) by providing causal evidence that decoding continues to constrain comprehension development in struggling readers beyond the early grades. They also align with the Reading is Language model (Snowling & Hulme, 2025), that emphasises cascading and reciprocal relations among reading components: our results provide support for an indirect effect of earlier decoding to later comprehension as postulated by the model.

Practically, the data indicate that sustained gains in comprehension among older, struggling readers can be achieved when interventions produce reliable improvements in word reading. This underlines the importance of explicit decoding instruction beyond the earliest grades and supports integrating decoding and comprehension activities so that children can repeatedly apply new decoding skills in meaningful contexts. The observed strengthening of comprehension at follow‐up—rather than decay—is encouraging for educators and policymakers because durable effects are rare in remediation studies and are critical for lasting academic impact.

### Limitations

One limitation of the study is the absence of repeated, multidimensional measures of oral language (e.g. grammatical or narrative skills), which limits our ability to comprehensively test language‐based mechanisms alongside decoding. Although we included a measure of taught vocabulary, this captures only a narrow aspect of oral language and may reflect individual differences in language learning as well as intervention effects. Future studies incorporating richer language measures are needed to clarify these mechanisms.

While randomisation produced comparable groups at baseline and models adjusted for pretest performance, we did not include additional covariates such as socio‐economic status. Although not required for unbiased estimation in an RCT, such variables could improve precision and allow tests of moderation. Finally, the follow‐up period was relatively short (4 months), reflecting practical constraints of the school year; longer‐term follow‐up is needed to determine whether gains persist, widen or attenuate over time.

## Conclusion

This study is among the first RCTs to embed a temporally lagged mediation design to test whether postintervention improvements in word‐level literacy contribute to later gains in reading comprehension in older struggling readers. RILL produced immediate and sustained improvements in word‐level literacy and reading comprehension, and the follow‐up comprehension effect was primarily accounted for by earlier gains in word‐level literacy at posttest, with little evidence of a remaining direct effect of the intervention on comprehension. Our findings provide support for a causal effect of word‐level literacy skills on later reading comprehension (as postulated by the Simple View of Reading (Gough & Tunmer, 1986) and Reading is Language model (Snowling & Hulme, 2025)). Practically, the results underscore the value of continuing explicit word‐reading instruction beyond the earliest grades and integrating it with meaningful text‐based and language‐rich activities to secure durable improvements in reading comprehension for struggling readers.

**Key points**

**What's known?**

Reading comprehension depends on both accurate word reading and oral language skills. Children who struggle with decoding often continue to experience difficulties with comprehension into the later primary years.

**What's new?**

This RCT demonstrates that a language‐rich, multicomponent literacy intervention produces immediate improvements in word reading, vocabulary and reading comprehension, with later comprehension gains mediated via improvements in both decoding and vocabulary.

**What's relevant?**

Our findings highlight that struggling readers in the later primary years continue to experience a decoding bottleneck but can still make meaningful progress. The intervention tested here is scalable, low‐cost and delivered by teaching assistants, underscoring its potential for wider educational practice and policy.

## Supporting information


**Appendix S1.** Word‐level literacy invariance model fit results.
**Table S1.** Configural, Metric and Scalar Model Fit Estimates Testing Word‐level Literacy Invariance between Pretest and Posttest.
**Table S2.** Configural, Metric and Scalar Model Fit Estimates Testing Word‐level Literacy Invariance between Pretest and Follow‐up.
**Appendix S2.** Reading comprehension invariance model fit results.
**Table S3.** Configural, Metric and Scalar Model Fit Estimates Testing Reading Comprehension Invariance between Pretest and Posttest.
**Table S4.** Configural, Metric and Scalar Model Fit Estimates Testing Reading Comprehension Invariance between Pretest and Follow‐up.
**Appendix S3.** Vocabulary invariance model fit results.
**Table S5.** Configural, Metric and Scalar Model Fit Estimates Testing Vocabulary Invariance between Pretest and Posttest.
**Table S6.** Configural, Metric and Scalar Model Fit Estimates Testing Vocabulary Invariance between Pretest and Follow‐up.

## Data Availability

The data that support the findings of this study are openly available in OSF at: https://osf.io/5vmqj/?view_only=92f4538009ca4657ab08975d03b364de.
