# Supplementary material for: Decoding and vocabulary improvements mediate sustained gains in reading comprehension: Evidence from a randomised controlled trial of a multicomponent reading intervention
Source: J Child Psychol Psychiatry. 2026 Apr 30;67(9):1562–75. doi: 10.1111/jcpp.70159 (PMC13450350; doi:10.1111/jcpp.70159)
Supplement: Supplementary file 1 [file JCPP-67-1562-s001.docx]

**Decoding and vocabulary improvements mediate sustained gains in reading comprehension: Evidence from a randomised controlled trial of a multi-component reading intervention**

**Supporting Information**

**Appendix S1. Word-level literacy invariance model fit results**

**Table S1.**

*Configural, Metric, and Scalar Model Fit Estimates Testing Word-level Literacy Invariance between Pretest and Posttest*

|  | $\chi^{2}$ | *df* | RMSEA | SRMR | CFI | TLI | $\chi_{\Delta}^{2}$ | $\Delta_{df}$ |
| --- | --- | --- | --- | --- | --- | --- | --- | --- |
| Full configural invariance | 41.00* | 24 | .050 | .074 | .99 | .98 | - | - |
| Full metric invariance | 43.08* | 27 | .046 | .084 | .99 | .98 | 2.08^n.s.^ | 3 |
| Full scalar invariance | 45.85 | 31 | .041 | .082 | .99 | .99 | 2.77^n.s.^ | 4 |

RMSEA = root mean square oferror approximation. 90% CI = 90% confidence intervals for RMSEA. SRMR = standardised root mean square residual. CFI = comparative fitindex. TLI = Tucker-Lewis index. ****p* < .001, ** *p* < .01, **p* < .05.

**Table S2.**

Configural, Metric, and Scalar Model Fit Estimates Testing Word-level Literacy Invariance between Pretest and Follow-up

|  | $\chi^{2}$ | *df* | RMSEA | SRMR | CFI | TLI | $\chi_{\Delta}^{2}$ | $\Delta_{df}$ |
| --- | --- | --- | --- | --- | --- | --- | --- | --- |
| Full configural invariance | 39.09* | 24 | .047 | .086 | .99 | .98 | - | - |
| Full metric invariance | 40.23* | 27 | .042 | .096 | .99 | .98 | 1.14^n.s.^ | 3 |
| Full scalar invariance | 42.19 | 31 | .036 | .094 | .99 | .99 | 1.96^n.s.^ | 4 |

RMSEA = root mean square oferror approximation. 90% CI = 90% confidence intervals for RMSEA. SRMR = standardised root mean square residual. CFI = comparative fitindex. TLI = Tucker-Lewis index. ****p* < .001, ** *p* < .01, **p* < .05

**Appendix S2. Reading comprehension invariance model fit results**

**Table S3.**

*Configural, Metric, and Scalar Model Fit Estimates Testing Reading Comprehension Invariance between Pretest and Posttest*

|  | $\chi^{2}$ | *df* | RMSEA | SRMR | CFI | TLI | $\chi_{\Delta}^{2}$ | $\Delta_{df}$ |
| --- | --- | --- | --- | --- | --- | --- | --- | --- |
| Full configural invariance | 35.62* | 23 | .045 | .087 | .98 | .97 | - | - |
| Partial metric invariance^†^ | 35.62* | 24 | .045 | .087 | .98 | .98 | 0^n.s.^ | 1 |

^†^Partial metric invariance was established by freeing the loadings at posttest for Passages 2, 3, and 4. RMSEA = root mean square oferror approximation. 90% CI = 90% confidence intervals for RMSEA. SRMR = standardised root mean square residual. CFI = comparative fitindex. TLI = Tucker-Lewis index. ****p* < .001, ** *p* < .01, **p* < .05.

**Table S4.**

Configural, Metric, and Scalar Model Fit Estimates Testing Reading Comprehension Invariance between Pretest and Follow-up

|  | $\chi^{2}$ | *df* | RMSEA | SRMR | CFI | TLI | $\chi_{\Delta}^{2}$ | $\Delta_{df}$ |
| --- | --- | --- | --- | --- | --- | --- | --- | --- |
| Full configural invariance | 42.04** | 22 | .057 | .088 | .97 | .95 | - | - |
| Partial metric invariance^†^ | 42.04** | 22 | .057 | .088 | .97 | .95 | 0^n.s.^ | 1 |

^†^Partial metric invariance was established by freeing the loadings at follow-up for Passages 2, 3, and 4. RMSEA = root mean square oferror approximation. 90% CI = 90% confidence intervals for RMSEA. SRMR = standardised root mean square residual. CFI = comparative fitindex. TLI = Tucker-Lewis index. ****p* < .001, ** *p* < .01, **p* < .05.

**Appendix S3. Vocabulary invariance model fit results**

**Table S5.**

*Configural, Metric, and Scalar Model Fit Estimates Testing Vocabulary Invariance between Pretest and Posttest*

|  | $\chi^{2}$ | *df* | RMSEA | SRMR | CFI | TLI | $\chi_{\Delta}^{2}$ | $\Delta_{df}$ |
| --- | --- | --- | --- | --- | --- | --- | --- | --- |
| Full configural invariance | 20.69 | 12 | .052 | .072 | .98 | .97 | - | - |
| Full metric invariance | 21.98 | 14 | .046 | .081 | .99 | .98 | 1.81^n.s.^ | 1 |
| Full scalar invariance | 25.28 | 17 | .043 | .088 | .98 | .98 | 3.30^n.s.^ | 3 |

RMSEA = root mean square oferror approximation. 90% CI = 90% confidence intervals for RMSEA. SRMR = standardised root mean square residual. CFI = comparative fitindex. TLI = Tucker-Lewis index.

**Table S6.**

Configural, Metric, and Scalar Model Fit Estimates Testing Vocabulary Invariance between Pretest and Follow-up

|  | $\chi^{2}$ | *df* | RMSEA | SRMR | CFI | TLI | $\chi_{\Delta}^{2}$ | $\Delta_{df}$ |
| --- | --- | --- | --- | --- | --- | --- | --- | --- |
| Full configural invariance | 27.26* | 13 | .063 | .076 | .98 | .96 | - | - |
| Full metric invariance | 29.07* | 14 | .062 | .088 | .98 | .96 | 1.14^n.s.^ | 1 |
| Full scalar invariance | 30.12 | 17 | .053 | .093 | .98 | .97 | 1.05^n.s.^ | 3 |

RMSEA = root mean square oferror approximation. 90% CI = 90% confidence intervals for RMSEA. SRMR = standardised root mean square residual. CFI = comparative fitindex. TLI = Tucker-Lewis index. ****p* < .001, ** *p* < .01, **p* < .05
